# Hypoxia Is a Critical Parameter for Chondrogenic Differentiation of Human Umbilical Cord Blood Mesenchymal Stem Cells in Type I/III Collagen Sponges

**DOI:** 10.3390/ijms18091933

**Published:** 2017-09-08

**Authors:** Tangni Gómez-Leduc, Mélanie Desancé, Magalie Hervieu, Florence Legendre, David Ollitrault, Claire de Vienne, Michel Herlicoviez, Philippe Galéra, Magali Demoor

**Affiliations:** 1 Normandie Université, Université de Caen Normandie, Laboratoire Microenvironnement Cellulaire et Pathologies (MILPAT), équipe Microenvironnement des Pathologies Dégénératives et Fibrotiques (MIPDF), EA 4652/BIOTARGEN EA 7450, UFR Santé, 14032 Caen, France; tangnigl@free.fr (T.G.-L.); melanie359@hotmail.fr (M.D.); magalie.hervieu@gmail.com (M.H.); florence.legendre@unicaen.fr (F.L.); david.ollitrault@gmail.com (D.O.); magali.demoor@unicaen.fr (M.D.); 2 Service de Gynécologie-Obstétrique et Médecine de la Reproduction, CHU Caen, 14033 Caen, France; devienne-c@chu-caen.fr (C.d.V.); michel.herlicoviez@unicaen.fr (M.H.)

**Keywords:** mesenchymal stem cells, umbilical cord blood, cartilage tissue engineering, hypoxia, chondrogenesis

## Abstract

Umbilical cord blood (UCB) is an attractive alternative to bone marrow for isolation of mesenchymal stem cells (MSCs) to treat articular cartilage defects. Here, we set out to determine the growth factors (bone morphogenetic protein 2 (BMP-2) and transforming growth factor-β (TGF-β1)) and oxygen tension effects during chondrogenesis of human UCB-MSCs for cartilage engineering. Chondrogenic differentiation was induced using 3D cultures in type I/III collagen sponges with chondrogenic factors in normoxia (21% O_2_) or hypoxia (<5% O_2_) for 7, 14 and 21 days. Our results show that UCB-MSCs can be committed to chondrogenesis in the presence of BMP-2+TGF-β1. Normoxia induced the highest levels of chondrocyte-specific markers. However, hypoxia exerted more benefit by decreasing collagen X and matrix metalloproteinase-13 (MMP13) expression, two chondrocyte hypertrophy markers. However, a better chondrogenesis was obtained by switching oxygen conditions, with seven days in normoxia followed by 14 days in hypoxia, since these conditions avoid hypertrophy of hUCB-MSC-derived chondrocytes while maintaining the expression of chondrocyte-specific markers observed in normoxia. Our study demonstrates that oxygen tension is a key factor for chondrogenesis and suggests that UBC-MSCs 3D-culture should begin in normoxia to obtain a more efficient chondrocyte differentiation before placing them in hypoxia for chondrocyte phenotype stabilization. UCB-MSCs are therefore a reliable source for cartilage engineering.

## 1. Introduction

Human mesenchymal stem cells (hMSCs) are multipotent cells able to differentiate into various cell types, including chondrocytes [1]. MSCs are therefore considered suitable for cartilage tissue engineering. MSCs were originally isolated from bone marrow, but were later found and isolated from numerous tissues [2]. However, in bone-marrow-derived mesenchymal stem cells (BM-MSCs), proliferation and differentiation potential towards the chondrocyte phenotype decrease with the age of the donor [3,4]. Current research is focusing on alternative sources of MSCs that have an inherent capacity to produce a hyaline articular cartilage extracellular matrix. Umbilical cord blood (UCB) is an attractive alternative source compared with BM-MSCs and for treating articular cartilage defects [5]. Thus, UCB-MSCs have an ontogenetically younger origin and therefore are not age-dependent. Furthermore, UCB-MSCs can be considered as an alternative MSC source given their high chondrogenic potential [6,7,8].

The in vitro process for chondrogenic differentiation of MSCs for tissue engineering requires three-dimensional (3D) tissue culture with the addition of growth factors to serum-free chondrogenic medium [1]. MSCs can be seeded at high cell density in pellet culture or within matrix scaffolds to mimic the mesenchymal condensation observed during embryonic chondrogenesis and to promote cell-cell contacts [9]. Numerous biomaterials based on natural or synthetic polymers have been explored as scaffolds to be used for cartilage engineering. Natural polymers include alginate, collagen, chitosan, fibrin, cellulose and others, in the form of hydrogels, sponges or fibrous meshes [2]. Among the different biomaterials available, our team uses type I/III collagen sponges for their chondrogenic potential and their compatibility with clinical practice for cartilage engineering strategies [10,11,12,13]. This biomaterial allows attachment of cells and the expression of chondrocyte-specific markers when cells are seeded in hypoxia in the presence of bone morphogenetic protein 2 (BMP-2) [12]. In addition, this collagen scaffold is biodegradable and is made with type I atelocollagen to remove the antigenic determinants. This collagen isotype is chemically crosslinked to obtain a 3D scaffold with good mechanical and thermal stability, an important characteristic for its use in cell therapy.

The use of growth factors is an important feature of successful chondrogenic differentiation. Transforming growth factor-β (TGF-β) super-family, in particular TGF-β1 and bone morphogenetic proteins (BMPs), are the most well-known factors for in vitro chondrogenic differentiation. The requirement of growth factors for the induction of chondrogenesis in MSCs is source-specific. For example, in cultures supplemented with TGF-β3, BM-MSCs display a gene expression profile more similar to mature cartilage during chondrogenic induction than adipose tissue-derived stem cells (ADSCs) [14]. The lower chondrogenic induction rate with TGF-β3 in ADSCs is probably due to the reduced expression of TGF-β receptors in these cells [15]. However, BMP-6 treatment induces the expression of the TGFβ-receptor-I and a chondrogenic response to TGF-β3 [15]. Nevertheless, only a limited number of studies have been performed with hUCB-MSCs for cartilage tissue engineering strategies [6,16,17,18,19]. In the literature, chondrogenic differentiation of hUCB-MSCs is generally induced using TGF-β1 or TGF-β3 in monolayer or pellet cultures [5,7,8,20,21]. To the best of our knowledge, only two studies have demonstrated that BMP-2 induces the differentiation of UCB-MSCs into chondrocytes [22,23]. Therefore, further studies are needed to establish the growth factor requirements for successfully inducing hUCB-MSCs chondrogenic differentiation.

In addition to growth factors, oxygen tension plays an important role in controlling the proliferation and differentiation of MSCs into chondrocytes. Articular cartilage is an avascular connective tissue in which oxygen is supplied to the chondrocytes by diffusion from the synovial fluid, with tension ranging from 1% O_2_ in the deep zones to 10% O_2_ at the surface [2]. Chondrocytes normally maintain homeostasis of articular cartilage through a limited turn-over of extracellular matrix (ECM) components. The maturation of chondrocytes is arrested in articular cartilage, and their differentiation towards a terminal hypertrophic phenotype is prevented. Expression of cartilage hypertrophy markers, e.g., type X collagen, matrix metalloproteinase-13 (MMP-13) and alkaline phosphatase in MSCs undergoing chondrogenesis raises concerns for MSC tissue engineering applications, because hypertrophy results in apoptosis and ossification [24]. The challenge of using MSCs as a cell source for articular cartilage tissue engineering is to prevent the MSC-derived chondrocytes from undergoing hypertrophic maturation. To mimic the physiological environment of chondrocytes, cell cultures must be performed under low oxygen tension (hypoxia). However, the response of MSCs at low oxygen tension is controversial in regard to chondrogenic differentiation. Low oxygen tension has been reported to support or inhibit chondrogenic differentiation [25,26,27,28,29]. However, there seems to be a consensus that hypoxia impedes terminal differentiation of MSCs during the in vitro chondrogenic differentiation of MSCs [30]. Therefore, hypoxia may be a useful tool for improving the quality-control during chondrogenic differentiation of MSCs. However, little is known about the effect of hypoxia on hUCB-MSCs during chondrogenic differentiation [17,31].

We have previously demonstrated that hUCB is a source of MSCs, able to commit to the chondrogenic lineage after two weeks of culture in the presence of BMP-2 and TGF-β1 in normoxia [6]. Further, sub-cutaneous implantation of hUCB-MSC-derived neo-cartilage constructs in nude mice after chondrogenic differentiation in vitro does not induce ECM mineralization [6]. Here, we examined the effects of BMP-2 (50 ng/mL) and of TGF-β1 (10 ng/mL) individually and together during hUCB-MSC chondrogenic differentiation using 3D cultures in normoxia (21% O_2_), in hypoxia (<5% O_2_), and with a combination of the two oxic conditions. Our results show that the hUCB-MSCs can commit to the chondrogenic pathway with slight variations according to the growth factor or oxygen tension used. Chondrogenic differentiation and endochondral ossification were assessed based on observed mRNA and protein levels. We demonstrated that modifying the oxygen conditions during differentiation limits hypertrophy of hUCB-MSC-derived chondrocytes and maintains or only slightly decreases the expression of the chondrocyte phenotype markers.

## 2. Results

### 2.1. Effects of Bone Morphogenetic Protein 2 (BMP-2) and/or Transforming Growth Factor-β (TGF-β1) on the Amounts of mRNA Encoding Cartilage-Specific Markers during Chondrogenesis of Human Umbilical Cord Mesenchymal Stem Cells (hUCB-MSCs)

We evaluated the effects of BMP-2 and/or TGF-β1 as well as oxygen tension on the differentiation of hUCB-MSCs into chondrocytes. For the chondrogenesis induction analysis, cells were seeded in collagen sponge scaffold at P4 and cultured in the presence or in the absence of the two chondrogenic factors during 7, 14 and 21 days in normoxia and/or hypoxia.

To characterize the differentiation status of UCB-MSCs, the mRNA amounts of several proteins, indicators of the differentiated, dedifferentiated and hypertrophic chondrocytes, were quantified. In parallel, the chondrocyte characteristic markers were also evaluated: type II collagen, aggrecan and *SOX-9* (SRY (**s**ex-determining **r**egion of the Y chromosome)-box-9), because this transcription factor positively regulates their expression [32]. Furthermore, we also evaluated the mRNA levels of transcripts encoding two type II collagen isoforms, prechondrogenic type IIA collagen (pre-chondrocyte and MSCs) and mature type IIB collagen (chondrocytes).

**Figure 1:**
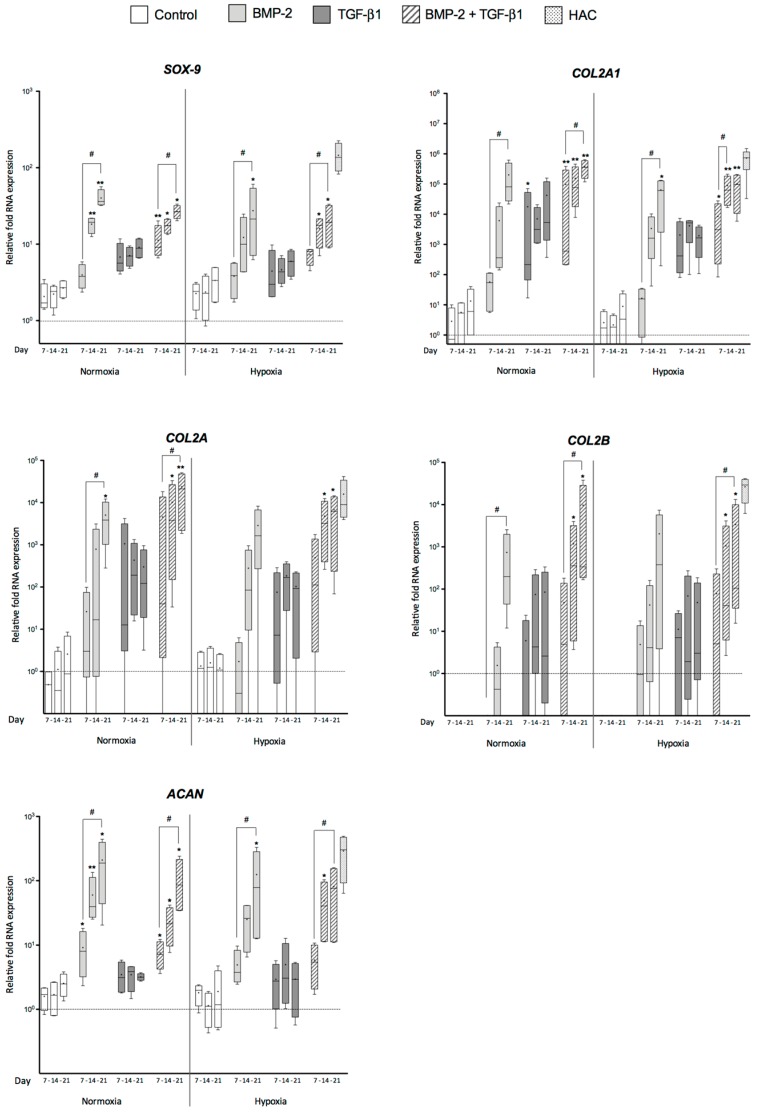
Effect of growth factors and oxygen tension on the gene expression of cartilage-specific markers during chondrogenic differentiation of human umbilical cord mesenchymal stem cells. Human umbilical cord mesenchymal stem cells (hUCB-MSCs) were cultured in type I collagen sponges for 7, 14 and 21 days in normoxia or in hypoxia, in the absence (untreated), or in the presence of 50 ng/mL of bone morphogenetic protein 2 (BMP-2), or 10 ng/mL of transforming growth factor-β (TGF-β1), or both BMP-2 and TGF-β1 (BMP-2+TGF-β1). Real-time RT-PCR analysis of relative mRNA expression of the indicated genes (*SOX-9* (sex-determining region of the Y chromosome)-box-9), *COL2A1*, *COL2A*, *COL2B* and *ACAN*) is shown. Gene expression was normalized to the endogenous reference gene, ribosomal protein L13a (*RPL13a*). Results are expressed as relative mRNA expression and data obtained were compared with undifferentiated hUCB-MSCs in monolayer culture (----) set at the arbitrary unit of 1. Hyaline articular cartilage (HAC) mRNAs were used as positive control. Box plots (least to greatest values, median (line in box) and mean (+)) show data derived from four donors in triplicate. Statistical analysis was performed using the non-parametric Kruskal–Wallis test to estimate fold change among samples. * *p* < 0.05, ** *p* < 0.01. The Friedman test was used to determine differences between time points. ^#^ *p* < 0.05. Comparison of the differences between normoxia and hypoxia was performed using the 2-tailed Mann–Whitney U test.

Regarding makers specific to chondrocytes (*SOX-9*, *ACAN*, *COL2A1*, *COL2A* and *COL2B*), hUCB-MSCs cultured in collagen scaffold without growth factors exhibited only a slight increase in the mRNA steady-state levels of *SOX-9*, *ACAN*, *COL2A1* and *COL2A*, but no type IIB collagen mRNA was detected (Figure 1). In contrast, there was a significant increase in the mRNA levels of *COL2A1* and its isoforms, *ACAN* and *SOX-9* in hUCB-MSCs cultured in collagen scaffolds in the presence of growth factors compared with undifferentiated cells in normoxia as well as in hypoxia (Figure 1). hUCB-MSCs in 3D culture treated with BMP-2 showed higher levels of *SOX-9* mRNA than untreated cells. The addition of BMP-2 alone or associated with TGF-β1 induced a continuous and strong increase over time in normoxia and in hypoxia. The relative mRNA levels of *SOX-9* after three weeks in the presence of BMP-2, alone or associated with TGF-β1, were, respectively, 40.2 and 26.8 in normoxia compared with undifferentiated cells, but 27.3 and 19.2 in hypoxia (Figure 1). There were no significant differences between TGF-β1-treated cells and controls without growth factors at each time point during culture. The treatment of cells with BMP-2 alone in normoxia at 21 days provided the closest chondrocyte-specific mRNA levels to the hyaline articular cartilage (HAC) control.

BMP-2 and/or TGF-β1 differentially stimulated the expression of *COL2A1* and its isoforms in hUCB-MSCs cultured in collagen scaffolds. The addition of BMP-2 alone or TGF-β1 alone improved the expression of the various type II collagen mRNAs, with a greater increase for the BMP-2 treatment compared with TGF-β1, regardless of the oxygen tension. Furthermore, in normoxia, BMP-2 induced a relative transcript abundance, which was 1.8 and 3.1 times higher compared with hypoxia for *COL2A1* and *COL2A* mRNA levels at Day 21, respectively. However, due to donor-specific responses to BMP-2, the difference between untreated and treated hUCB-MSCs in 3D culture was only significant for *COL2A1* at 21 days in hypoxia and *COL2A* at 21 days in normoxia. The addition of TGF-β1 caused an increase in the expression of the various type II collagen mRNAs at seven days, but mRNA levels remained relatively stable at 14 and 21 days, and low compared with BMP-2 alone in normoxia and hypoxia. The combination of both BMP-2 and TGF-β1 had a stronger effect. The association of growth factors induced the expression of all forms of type II collagen mRNAs during culture time, in normoxia and hypoxia, reaching levels close to the HAC control. The relative mRNA expressions of *COL2A1*, *COL2A* and *COL2B* in normoxia were, respectively, 3.7, 3.5, and 2.9 times higher compared with hypoxia at 21 days of chondrogenic differentiation.

Regarding aggrecan, the collagen scaffold culture with TGF-β1 alone did not significantly increase *ACAN* expression, whereas all conditions including BMP-2 had a stimulating effect (Figure 1). The addition of TGF-β1 alone displayed *ACAN* levels close to untreated hUCB-MSCs grown in 3D culture with, respectively, 3–5-fold and 1.6–2.5-fold change over the 21 days of differentiation. The relative mRNA expressions of *ACAN* at the end of the culture in the presence of BMP-2 alone or associated with TGF-β1, were, respectively, 209.4 and 111.7 in normoxia compared with undifferentiated cells, whereas they were 124.4 and 80.4 in hypoxia (Figure 1). The expression level of *ACAN* in differentiated chondrocytes in the presence of BMP-2 in normoxia at 21 days was close to that of the HAC control (289-fold). These results suggest that the addition of BMP-2 is extremely important for inducing *ACAN* expression during the differentiation of hUCB-MSCs into chondrocytes.

In summary, hUCB-MSCs commit to the chondrogenic lineage, but growth factors are essential for differentiation regardless of oxygen tension. The highest expression levels for chondrocyte-specific markers (*SOX-9*, *ACAN*, *COL2A1*, *COL2A* and *COL2B*) were obtained after 21 days of culture in the presence of BMP-2 alone or with both BMP-2 and TGF-β1. In addition, we noticed a trend of higher mRNA expression in normoxia than in hypoxia.

### 2.2. Effects of BMP-2 and/or TGF-β1 on the Levels of mRNA Encoding Non-Cartilage Markers during Chondrogenic Commitment of hUCB-MSCs

We then sought to determine whether the chondrogenic differentiation conditions also induce the expression of non-cartilage markers in hUCB-MSCs-derived chondrocytes such as: *COL1A1* (dedifferentiated chondrocytes); *COL10A1*, *MMP-1*, and *MMP-13* (hypertrophic chondrocytes); and runt-related transcription factor-2 (*RUNX-2*) and *OSTEOCALCIN* (osteoblasts).

**Figure 2:**
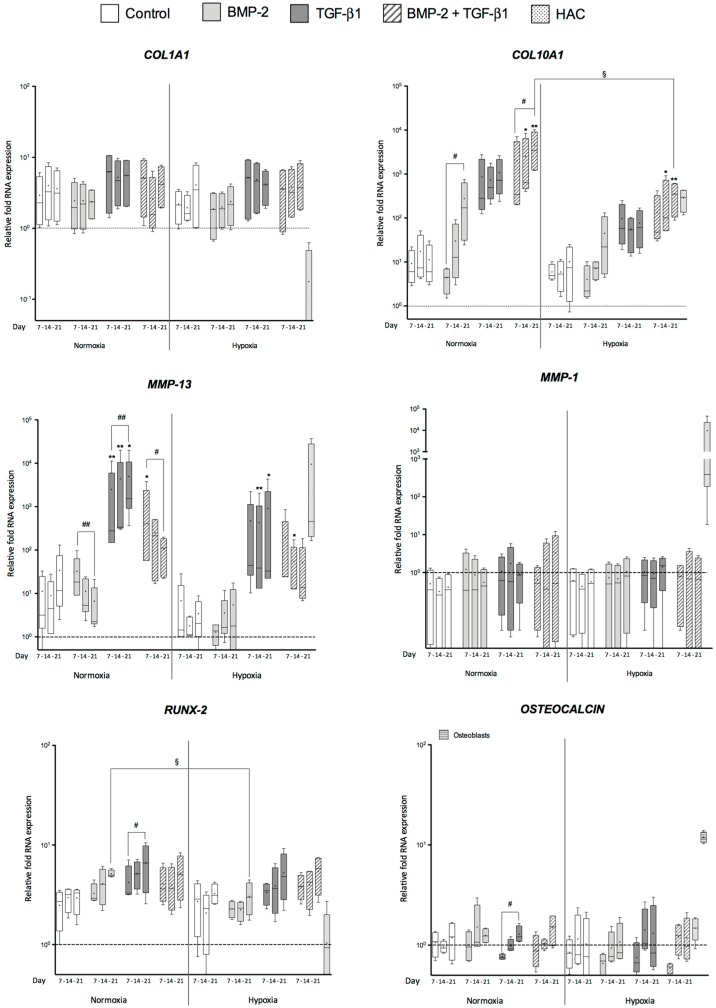
Effect of culture conditions on the gene expression of non-cartilage markers during chondrogenic differentiation of human umbilical cord mesenchymal stem cells. Human umbilical cord mesenchymal stem cells (hUCB-MSCs) were cultured in type I collagen sponges for 7, 14 and 21 days in normoxia or in hypoxia, in the absence (untreated), or in the presence of 50 ng/mL of BMP-2, or 10 ng/mL of TGF-β1, or both BMP-2 and TGF-β1 (BMP-2 + TGF-β1). Real-time RT-PCR analysis of relative mRNA expression of the indicated genes (*COL1A1*, *COL10A1*, *MMP-1*, *MMP-13*, runt-related transcription factor-2 (*RUNX-2*) and *OSTEOCALCIN*) is shown. Gene expression was normalized to the endogenous reference gene, *RPL13a*. Results are expressed as relative mRNA expression and data obtained were compared with undifferentiated hUCB-MSCs in monolayer culture (----) set at the arbitrary unit of 1. Hyaline articular cartilage (HAC) mRNAs were used as positive control in all the panels whereas osteoblast mRNAs were used as positive control only for *OSTEOCALCIN* RT-qPCR (box-plot on the extreme right part of the panel). Box plots (least to greatest values, median (line in box) and mean (+)) represent data derived from four donors in triplicate. Statistical analysis was performed using the non-parametric Kruskal–Wallis test to estimate fold change among samples. * *p* < 0.05, ** *p* < 0.01. The Friedman test was used to determine differences between time points. ^#^ *p* < 0.05, ^##^ *p* < 0.01. Comparison of differences between normoxia and hypoxia was performed using the two-tailed Mann–Whitney U test. (§): ^§^ *p* < 0.05.

In collagen scaffold cultures, *COL1A1* mRNA levels remained relatively stable regardless of the growth factor treatment in normoxia and in hypoxia (Figure 2). However, the expression of *COL1A1* in monolayers of undifferentiated cells and 3D cultures remained higher than the HAC levels.

Type X collagen and MMP-13 are two well-known markers of chondrocyte hypertrophy. They are generally associated with endochondral ossification and are normally absent in hyaline cartilage. Here, growth factors and oxygen tension influenced *COL10A1* mRNA expression during chondrogenic differentiation of hUCB-MSCs in 3D culture (Figure 2). The collagen sponge culture without growth factors induced a slight increase in *COL10A1* mRNA levels in normoxia and in hypoxia compared with undifferentiated cells. In normoxia, the addition of BMP-2 caused a significant increase in *COL10A1* mRNA between 7 and 21 days during chondrogenic differentiation compared with untreated cells. A similar tendency was observed for the TGF-β1 treatment. Moreover, after 14 and 21 days of chondrogenic differentiation in normoxia, *COL10A1* mRNA amounts were higher in cells treated with both BMP-2 and TGF-β1 than in untreated hUCB-MSCs. These data clearly show an inductive effect of the combination of BMP-2 and TGF-β1 in normoxia on the commitment of hUCB-MSCs to a hypertrophic state. However, at the end of the culture period, *COL10A1* expression was greatly and significantly decreased in hypoxia in the presence of both BMP-2 and TGF-β1 compared with normoxia. The relative *COL10A1* mRNA expression in the presence of BMP-2 and TGF-β1 at 21 days was 3580-fold higher in normoxia compared with the undifferentiated hUCB-MSCs, whereas it was only 350.8-fold higher in hypoxia. In addition, the level of type X collagen expression in the differentiated chondrocytes in hypoxia was close to that of the HAC control (286-fold more than undifferentiated hUCB-MSCs).

*MMP-13* expression was differentially regulated, depending on the growth factor used to induce chondrogenesis. The addition of BMP-2 alone, in normoxia, was associated with the lowest relative *MMP-13* mRNA expression, and a continuously decreasing expression was observed between 7 and 21 days, from 32.6- to 6.4-fold. On the contrary, TGF-β1 alone induced higher expression of *MMP-13* because as early as seven days in normoxia, levels increased during chondrogenic differentiation from 2490- to 4973-fold and in hypoxia from 469- to 908-fold. These data clearly show an inductive effect of TGF-β1 on the commitment of hUCB-MSCs to hypertrophic chondrocytes. The combination of both BMP-2 and TGF-β1 led to an intermediate *MMP-13* expression profile at Day 7 compared with BMP-2 alone and TGF-β1 alone. Interestingly, the BMP-2/TGF-β1 combination led to a decrease in *MMP-13* levels during chondrogenic differentiation in normoxia (from 1054 to 105) and in hypoxia (from 195 to 51). MMP-1, which specifically degrades type I, II and III collagens, did not seem to be regulated in any of the conditions, with and without growth factors. MMP-1 expression was lower in all groups compared to HACs. The unusual MMP-1 expression of HACs control may be due to the enzymatic treatment of cartilage biopsies.

hUCB-MSCs cultured in normoxic or in hypoxic conditions did not perform osteogenic differentiation in type I/III collagen sponges constructs since the steady-state amounts of osteocalcin mRNA were stable, close to HAC control. For *RUNX-2*, the mRNA amounts remained also stable and above HAC control. However, we noticed a significant and progressive increase in its expression with TGF-β1 alone during culture time.

**Figure 3:**
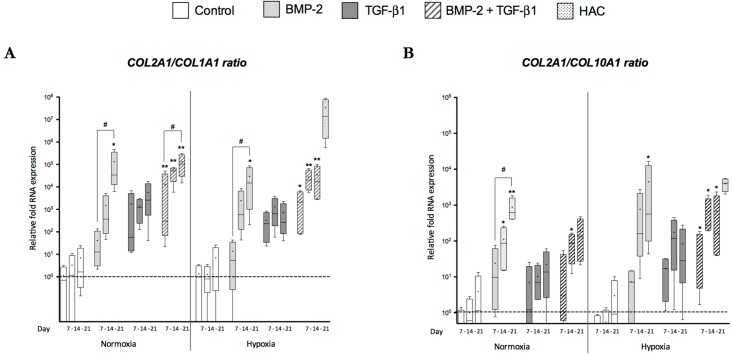
Effect of culture conditions on the *COL2A1* mRNA/*COL1A1* mRNA and *COL2A1* mRNA/*COL10A1* mRNA ratios during chondrogenic differentiation of human umbilical cord mesenchymal stem cells. The human umbilical cord mesenchymal stem cells (hUCB-MSCs) were incubated in the same experimental conditions as those described in the legends of Figure 2 and Figure 3, and gene expression analysis was performed in an identical manner. The functional index of the hUCB-MSCs induced in chondrocytes was determined by determining the ratios of *COL2A1* mRNA:*COL1A1* mRNA (**A**) and *COL2A1* mRNA:*COL10A1* mRNA (**B**). Statistical analysis was performed using the non-parametric Kruskal–Wallis test to estimate fold change among samples. * *p* < 0.05, ** *p* < 0.01. The Friedman test was used to determine differences between time points. ^#^ *p* < 0.05.

To consolidate our analyses, we evaluated the *COL2A1:COL1A1* ratio at steady-state mRNA levels (Figure 3A), which provides an indication of the differentiation status of chondrocytes and their functional index. The highest ratio values were obtained with BMP-2 alone and with its combination with TGF-β1 regardless of oxygen tension. In addition, there was an increase between Day 7 and Day 21. The phenotypic profile analysis was completed by evaluating of the ratio of type II collagen mRNA to type X collagen mRNA (Figure 3B). After seven days, the ratio values remained low regardless of growth factor treatment and oxygen tension. These ratios were higher at Day 14 in normoxia with BMP-2 alone and in hypoxia with BMP-2 and TGF-β1 together. Hypoxia provided ratio values for the combined BMP-2/TGF-β1 treatment that were equivalent or even higher than those observed in normoxia. These results suggest that oxygen tension affects the quality of the in vitro chondrogenesis of hUCB-MSCs.

Overall, these data provide evidence that hUCB-MSCs, cultured in a collagen scaffold in normoxia and in hypoxia, can commit to the chondrogenic lineage, with slight variations according to the growth factor and oxygen tension used. By combining BMP-2 and TGF-β1 in a 3D culture model, we obtained a significant induction of chondrogenic differentiation. The best differentiated phenotypic profile was observed after 21 days of culture, and showed synthesis of chondrocyte phenotype markers: type IIB collagen, aggrecan, and *SOX-9* This profile indicates effective chondrocyte differentiation. Nevertheless, hypertrophy of the chondrocytes was also enhanced by BMP-2 and TGF-β1, particularly in normoxia.

In contrast, hypoxia prevents terminal differentiation of hUCB-MSCs induced by the growth factors. Finally, low levels of *COL1A1* mRNA persist in all the culture conditions. The highest values of *COL2A1*:*COL1A1* and *COL2A1*:*COL10A1* mRNA ratios confirm that the most appropriate conditions for optimum chondrogenic differentiation is BMP-2 combined with TGF-β1 and that hypoxia is suitable for maintaining the differentiated chondrocyte phenotype.

### 2.3. Newly Synthesized Extracellular Matrix (ECM) Proteins Expressed by hUCB-MSCs Differentiating into Chondrocytes

Cells were harvested after 7, 14 and 21 days of differentiation for protein extraction and Western blot analysis to estimate the protein expression levels of type II collagen, a chondrocyte-specific maker, and of dedifferentiated and hypertrophic chondrocyte markers, respectively type I and X collagens.

**Figure 4:**
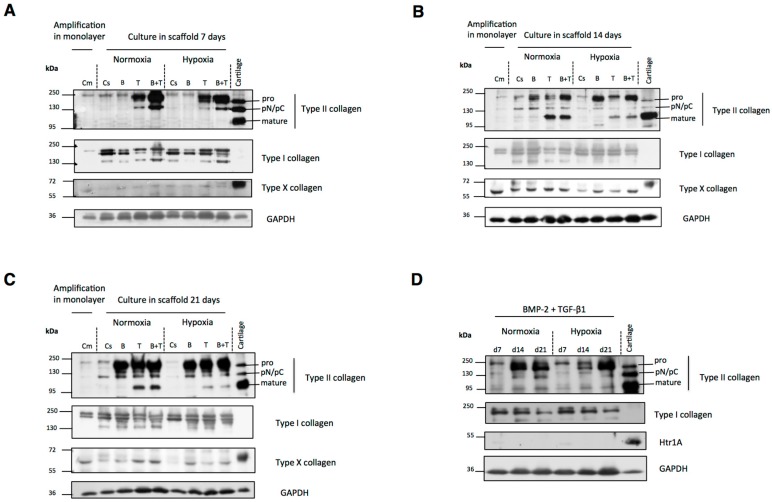
Western blot analysis of human umbilical cord mesenchymal stem cells differentiated into chondrocytes. Human umbilical cord mesenchymal stem cells (hUCB-MSCs) were cultured in type I collagen sponges for 7, 14 and 21 days in normoxia or in hypoxia, in the absence (Control), or in the presence of 50 ng/mL of BMP-2, or 10 ng/mL of TGF-β1, or both BMP-2 and TGF-β1. Representative images of banding patterns are shown. (**A**–**C**) Western blot analysis of type I/II/X collagens and glyceraldehyde-3-phosphate dehydrogenase (GAPDH) of undifferentiated hUCB-MSCs in monolayer (Cm), untreated hUCB-MSCs in 3D culture (Cs) and hUCB-MSCs in 3D culture incubated in chondrogenic medium containing BMP-2 (B) or TGF-β1 (T), or both BMP-2 and TGF-β1 (B+T) in normoxia or hypoxia after: 7 (**A**); 14 (**B**); and 21 (**C**) days. (**D**) Western blot analysis of type I/II collagens and GAPDH in chondrogenic differentiated cells incubated with BMP-2 and TGF-β1 in scaffold in normoxia and hypoxia after 7, 14 and 21 days. Cartilage protein extracts were used as positive controls in all the experiments.

As shown in Figure 4A, type II collagen expression after one week of chondrogenic differentiation depended on the growth factor treatments and oxygen tension. Undifferentiated (monolayer cultures) hUCB-MSCs, untreated cells and BMP-2-treated cells very weakly expressed type II procollagen in normoxia and in hypoxia. In contrast, TGF-β1 treatment increased the production of procollagen and pN/pC forms of type II collagen regardless of oxygen tension. The highest amount was obtained with both BMP-2 and TGF-β1. In addition, the synthesis of all the type II collagen maturation forms was higher in normoxia. However, the mature form of type II collagen was not detected regardless of culture conditions. After two weeks of chondrogenic differentiation without growth factors, the cells still weakly expressed procollagen and pN/pC form of type II collagen (Figure 4B). In the presence of BMP-2 alone, the cells synthesized pro and pN/pC forms of type II collagen. The cells incubated with TGF-β1 alone or in combination with BMP-2 synthesized the mature form of type II collagen (resulting from the cleavage of the N- and C-terminal propeptides of type II procollagen), with higher protein expression in normoxia. The amount of the mature form of type II collagen in the hUCB-MSCs differentiated into chondrocytes with TGF-β1 alone or in combination with BMP-2 in normoxia was practically comparable to the HAC control, thus confirming induction of a chondrocytic phenotype in these culture conditions. After 21 days of differentiation, all type II collagen forms showed the same band pattern observed at 14 days (Figure 4C).

Regarding the non-cartilage markers, type I procollagen expression was not modified during chondrogenesis induction, as attested by the unchanged procollagen band pattern at 7, 14 and 21 days (Figure 4A–C). However, the pN form of type I collagen was expressed less at 14 and 21 days in hypoxia than in normoxia. The type X collagen protein level was lower in hypoxia than in normoxia regardless of the growth factor used for chondrogenic differentiation of hUCB-MSCs, particularly after 14 and 21 days of culture (Figure 4B,C).

Type I collagen, which is characteristic of fibrocartilage but not of mature hyaline cartilage, seemed to be always present in all culture conditions at each time point. Figure 4D shows the type I and II collagen expression levels in the presence of BMP-2 and TGF-β1 in normoxia and in hypoxia in hUCB-MSCs during chondrogenic differentiation from Day 7 to Day 21. We observed for hUCB-MSCs differentiated both in normoxia or hypoxia that type I collagen decreased after treatment with BMP-2 and TGF-β1 during the 21 day culture period, whereas type II collagen synthesis increased. This decrease of type I collagen production may reflect the progressive stabilization of the chondrocytic phenotype. These results suggest a better functional differentiation index at 21 days, represented by the ratio of type II to type I collagens, an effect already observed at the mRNA level in normoxia and hypoxia (see Figure 3).

**Figure 5:**
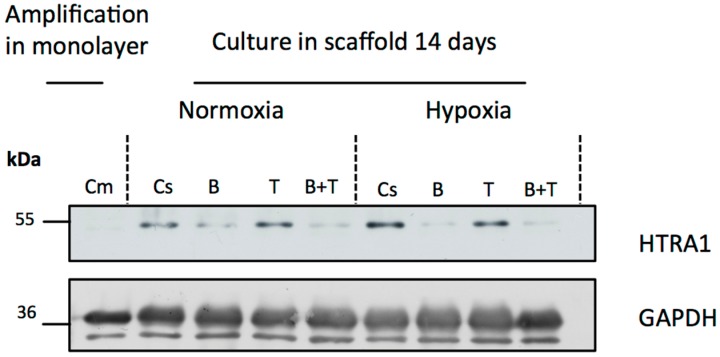
Western blot analysis of HtrA1 in human umbilical cord mesenchymal stem cells differentiated into chondrocytes. Human umbilical cord mesenchymal stem cells (hUCB-MSCs) were cultured in type I collagen sponges in normoxia or hypoxia, in the absence (untreated), or in the presence of 50 ng/mL of BMP-2, or 10 ng/mL of TGF-β1, or both BMP-2 and TGF-β1. Western blot analysis of HtrA1 and GAPDH in undifferentiated hUCB-MSCs in monolayer (Cm), untreated hUCB-MSCs in 3D culture (Cs) and hUCB-MSCs in 3D culture incubated in chondrogenic medium containing BMP-2 (B) or TGF-β1 (T), or both BMP-2 and TGF-β1 (B+T) in normoxia and hypoxia after 14 days.

Next, we investigated whether high-temperature requirement A serine peptidase 1 (HtrA1) serine protease (overexpressed in dedifferentiated osteoarthritic chondrocytes) was present and whether its expression was regulated by growth factors during the differentiation of hUCB-MSCs into chondrocytes. As shown in Figure 5, HtrA1 expression was not detected in undifferentiated cells amplified in monolayers. After 14 days of culture, untreated cells in 3D culture and cells treated with TGF-β1 alone expressed HtrA1 in normoxia and in hypoxia. In contrast, in all the conditions in which the cells were incubated in the presence of BMP-2, there was a decrease in HtrA1 expression compared with untreated cells in 3D culture. These results suggest that BMP-2 down-regulates the expression of HtrA1 in our culture model. Therefore, this combination is the most suitable for the synthesis of a cartilage matrix and its use for therapy. Moreover, with combined BMP-2 and TGF-β1 treatment, HtrA1 decreased progressively, becoming almost undetectable after 21 days of culture [33].

Western blot analyses confirmed our RT-qPCR results. In normoxia, the combination of both BMP-2 and TGF-β1 promoted the expression of type II and X collagens. The induction of differentiation in hypoxia may limit cell hypertrophy. Moreover, although TGF-β1 alone showed a high ability to induce the synthesis of type II collagen, it promoted also the expression of *MMP*-*13* and HtrA1. BMP-2 alone induced a later protein expression and did not promote the formation of mature type II collagen. Therefore, TGF-β1 or BMP-2 should not be used alone to induce chondrogenic differentiation. BMP-2 and TGF-β1 together were able to induce type II collagen synthesis at a high level, especially the mature form, and at the same time they limited the production of non-cartilage-specific *MMP-13*, HtrA1, and type I collagen markers in normoxia and hypoxia during chondrogenic differentiation.

### 2.4. Modification of Oxygen Tension Conditions during Differentiation towards the Chondrocyte Lineage Improves the Chondrocyte Phenotype

To benefit from the intrinsic effects of both normoxia and hypoxia, we modified the oxygen tension conditions during chondrogenic differentiation of hUCB-MSCs. For this purpose, we induced differentiation for seven days in normoxia followed by 14 days in hypoxia. The aim of these experiments was to initiate early differentiation in normoxia and then stabilize the chondrocyte phenotype under hypoxia, minimizing in fine the expression of the hypertrophy markers. The experiments were performed with hUCB-MSCs cultured only in 3D under combined BMP-2 and TGF-β1 treatments which were previously defined in the present study as the best conditions for chondrogenesis.

**Figure 6:**
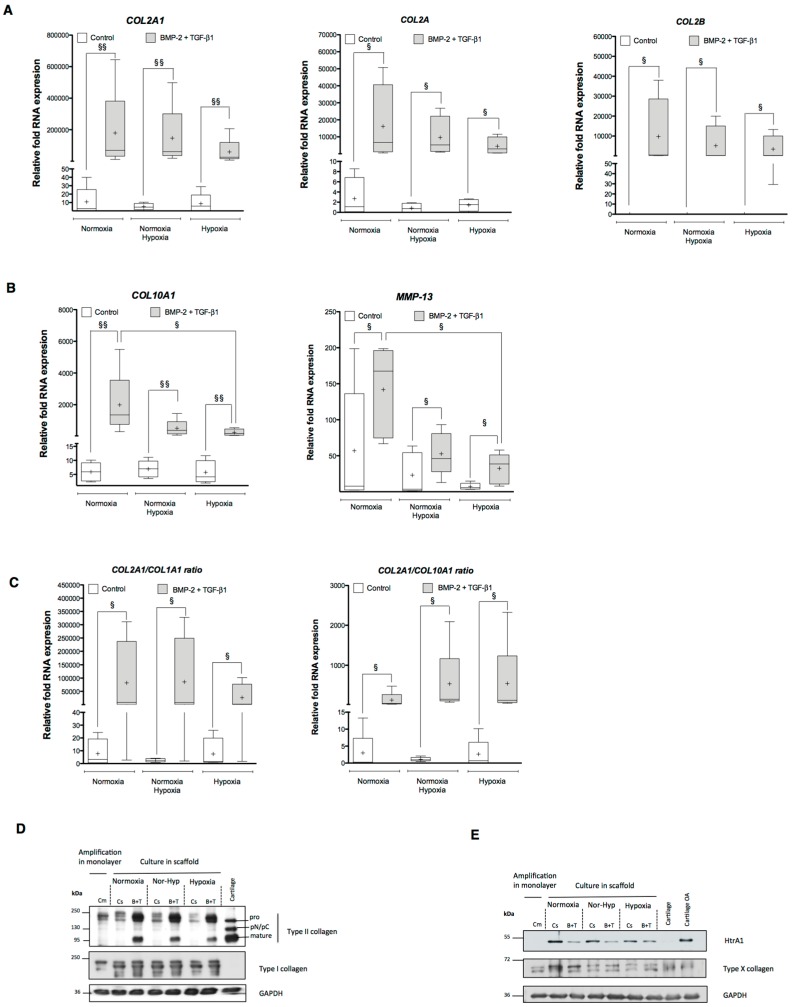
Effect of oxygen tension modification on the expression of cartilage-specific and non-cartilage markers during chondrogenic differentiation of human umbilical cord mesenchymal stem cells. Human umbilical cord mesenchymal stem cells (hUCB-MSCs) were cultured in type I collagen sponges for seven days in normoxia and then incubated for 14 days in hypoxia (Nor-Hyp). hUCB-MSCs differentiated into chondrocytes for 21 days either in normoxia or in hypoxia were used as control. Chondrogenic differentiation was induced with or without 50 ng/mL of BMP-2, and 10 ng/mL of TGF-β1. Real-time RT-PCR analysis of the relative mRNA expression of: chondrogenic markers (*COL2A1*, *COL2A* and *COL2B*) (**A**); and hypertrophic chondrocyte markers (*COL10A1* and *MMP-13*) (**B**). Gene expression was normalized to the endogenous reference gene, *RPL13a*. Results are expressed as relative mRNA expression and data obtained were compared with undifferentiated hUCB-MSCs cultured in monolayers set at the arbitrary unit of 1. (**C**) *COL2A1* mRNA/*COL1A1* mRNA and *COL2A1* mRNA/*COL10A1* mRNA ratios are presented. Box plots (least to greatest values, median (line in box) and mean (+)) represent data derived from four donors in triplicate. Statistical analysis was performed using the two-tailed Mann–Whitney U test. ^§^ *p* < 0.05, ^§§^ *p* < 0.01 (**A**–**C**). (**D**,**E**) Western blot analysis of type I/II/X collagens, HtrA1 and GAPDH in undifferentiated hUCB-MSCs in monolayer (Cm), untreated hUCB-MSCs in 3D culture (Cs) and hUCB-MSCs incubated in the chondrogenic medium containing BMP-2 and TGF-β1 (B+T).

When examining the effect of oxygen tension on the expression of markers specific to chondrocytes, we observed only a slight decrease in the relative mRNA expression of *COL2A1* compared with the cells differentiated in normoxia, from 179,204- to 146,794-fold (Figure 6A). However, the relative mRNA expression after modification of the oxygen tension remained above the levels observed in hypoxia (59,232). Other specific markers of articular cartilage including *COL2A* and *COL2B* also displayed the same tendency (Figure 6A). The change in oxygen tension during chondrogenic differentiation limits cell hypertrophy: *COL10A1* and *MMP-13* mRNA levels were much lower in hypoxia and in the modified oxygen tension conditions than in normoxia (Figure 6B). Relative *COL10A1* and *MMP-13* mRNA expression in hUCB-MSCs differentiated into chondrocytes in successive normoxia-hypoxia oxygen tension conditions was respectively 3.9 and 3.3 times lower than hUCB-MSCs cultured exclusively in normoxia, suggesting an inhibition of terminal differentiation in chondrocytes.

Furthermore, the *COL2A1* mRNA:*COL1A1* mRNA ratio was much higher in hUCB-MSCs previously differentiated in normoxia and thereafter incubated in hypoxia during chondrogenic differentiation than in the hypoxic condition alone (Figure 6C). The *COL2A1* mRNA:*COL10A1* mRNA ratio was also more beneficial in the successive normoxia/hypoxia conditions than in the exclusively normoxia condition (Figure 6C). These results suggest that low oxygen tension leads to the differentiation of mature chondrocytes and not to hypertrophic chondrocytes as demonstrated by the type II collagen mRNA ratios. These data confirm the importance of oxygen tension in hUCB-MSC differentiation into chondrocytes. The incubation of cells in hypoxia counteracts cell hypertrophy and provides better stabilization of the mature chondrocyte phenotype in vitro.

At the protein level, we obtained the same response in hUCB-MSC chondrogenic differentiation using the successive normoxia/hypoxia oxygen tension protocol. The protein study confirmed the results obtained at the mRNA level. Type II collagen showed an intermediate expression between normoxia and hypoxia alone the successive normoxia-hypoxia protocol (Figure 6D). Type I collagen expression in the hUCB-MSC undergoing chondrogenesis did vary according the different oxic conditions and the growth factor treatments (Figure 6D). In addition, type X collagen expression was lower in hypoxia or after successive normoxia-hypoxia incubation compared with the normoxia incubation (Figure 6E). Finally, the combination of BMP-2 and TGF-β1 in hypoxia also decreased protein expression of HtrA1 compared with untreated cells (Figure 6E).

The results obtained after normoxic chondrogenic differentiation of hUCB-MSCs followed by incubation in hypoxia led to a culture method combining the benefits of both oxygen tensions. The transition from normoxia to hypoxia during the differentiation limits cell hypertrophy by inhibiting the expression of type X collagen and *MMP-13*. Moreover, this strategy only slightly reduces the expression of type II collagen, which is still greater than that observed in hypoxia alone. This protocol significantly improves the chondrocyte phenotype, triggering early differentiation in normoxia and then stabilizing the chondrocyte phenotype in hypoxia by minimizing the expression of hypertrophy markers.

## 3. Discussion

In the present study, we set out to determine the optimal conditions for hUCB-MSC chondrocyte differentiation and thus the synthesis of HAC. To do so, we evaluated the response of hUCB-MSCs in the presence of BMP-2 and/or TGF-β1 in normoxia (21% O_2_) and hypoxia (<5% O_2_). Our results showed chondrogenic commitment of hUCB-MSCs cultured in collagen scaffolds, with slight variations according to the growth factor and oxygen tension used. Normoxia promoted higher expression of markers characteristic of the chondrocyte phenotype, but also enhanced chondrocyte hypertrophy. In contrast, hypoxia appeared to limit the expression of markers typical of mature chondrocytes, but prevented cell hypertrophy.

### 3.1. Cartilage-Specific Markers during Chondrogenesis of hUCB-MSCs

hUCB-MSC culture in collagen scaffolds in basal medium after 3 weeks of incubation was not sufficient to induce proper chondrogenic differentiation (e.g., type II collagen, aggrecan and *SOX-*9). Growth factors were clearly essential to attain chondrogenesis in MSCs in vitro regardless of oxygen tension. These results corroborate our previous data obtained after 2 weeks of culture of hUCB-MSCs in normoxia [6]. A previous study has shown that human BM-MSCs cultured in alginate beads under hypoxia leads to their differentiation into chondrocyte-like cells after seven days [34]. This discrepancy is probably due to cell type and/or the culture model.

According to the growth factors used to induce chondrogenic differentiation, we obtained different cell responses for the expression of type II collagen. Type II collagen constitutes more than 80% of normal articular cartilage ECM, providing the cartilaginous structure and tensile strength; its appearance is considered to be cartilage-specific. At the protein level, collagen II strongly induced after treatment of hUCB-MSCs with TGF-β1, which promotes the mature form of this collagen. Interestingly, in the presence of TGF-β1 alone or in association with BMP-2, the cells appeared to synthesize all the specific enzymes necessary for the removal of the propeptides (N- and C-propeptidases), which is a prerequisite for the maturation and correct assembly of collagen, and thus functional macromolecular ECM organization. The resulting mature type II collagen molecule, composed of N-telopeptide, C-telopeptide, and major triple helix parts, can assemble into heterotypic collagen fibrils with type IX and XI collagens to form typical cartilage collagen fibrils [35]. In contrast, the BMP-2 treatment alone induced lower expression of type II collagen and did not induce complete maturation of type II collagen regardless of the oxygen tension. We used BMP-2 at 50 ng/mL, which is a relatively low concentration compared with those reported in the literature, ranging from 10 to 500 ng/mL for chondrogenic MCS differentiation and chondrocyte redifferentiation [12,23]. An increase in BMP-2 concentration may possibly increase the chondrogenic differentiation potential, but may also have an impact on the expression of atypical markers of cartilage or induce osteogenic differentiation. For instance, a recombinant BMP-2 protein is known to induce both osteogenic and chondrogenic differentiation of MSCs in vitro [36,37,38,39] and is a well-established osteoinductive molecule currently used for clinical practice with supraphysiological doses of 0.4–1.5 mg/mL [40]. Furthermore, the addition of BMP-2 and TGF-β1 together revealed a stronger effect at the transcript and protein levels for type II collagen expression. These results are in agreement with other studies showing that TGF-β1 or TGF-β3 alone are able to induce chondrogenesis and that adding a second bioactive molecule such as BMP-2 improves the differentiation of MSCs in vitro [41,42,43,44]. The effect of growth factors on the expression of type II collagen can be attributed to a similar molecular mechanism of action for both growth factors. BMP-2 and TGF-β1 are ligands of the TGF-β superfamily and act by binding to type II-specific receptors recruiting the corresponding type I receptor, ultimately leading to phosphorylation of the S_mothers against Dpp (Smad)-receptor protein. Although the signaling of BMP-2 is mainly mediated through Smad 1/5/8 and that of TGF-β1 via SMAD 2/3, it is known that interactions between the two pathways are carried out downstream of Smad 4.

Aggrecan constitutes 80–90% of proteoglycans in articular cartilage. Proteoglycan aggregates are responsible for providing compressive and elastic strength to articular cartilage as well as regulating fluid in the cartilage matrix. *ACAN* synthesis was favored in conditions that included BMP-2 in the basal culture medium and its expression gradually increased during differentiation, whereas when the cells were treated with TGF-β1 alone, *ACAN* expression was comparable to untreated cells in collagen scaffolds. This suggests that aggrecan expression is modulated during chondrogenesis by BMP-2 and may play a role in this commitment. Similarly, for BM-MSCs under chondrogenic conditions in pellet cultures, *ACAN* expression is higher in the presence of BMP-2 (50 ng/mL) compared with TGF-β3 (10 ng/mL) [45]. Furthermore, regarding hUCB-MSCs cultured in micromass, treatment with BMP-2 (500 ng/mL) increases mRNA expression of aggrecan and its expression is greater than when the cells are exposed to BMP-6 (500 ng/mL) [23].

Thus, in our study, the concomitant addition of BMP-2 and TGF-β1 constitutes the best treatment, allowing the expression of the two major ECM components of articular cartilage: the mature form of type II collagen and aggrecan. To investigate growth factor requirements, we also performed chondrogenic differentiation with TGF-β3 (10 ng/mL) alone or in combination with BMP-2 with similar results as those obtained with TGF-β1 in regard to mRNA and protein expression for chondrocyte-specific and non-chondrocyte markers [33]. Consistent with our findings, Mwale et al. also showed the similarity of the results obtained in the presence of TGF-β1 or TGF-β3 during in vitro chondrogenesis [46].

### 3.2. Atypical Markers of Cartilage during Chondrogenesis of hUCB-MSCs

Cartilage therapy cannot be applied to hypertrophic cells, because this state involves chondrocyte apoptosis and ossification of the ECM. In other words, chondrocyte phenotypic stability is required for the cells to produce a functional hyaline ECM. In our study, both oxygen tension conditions induced chondrogenesis. However, there were some subtle differences between the two conditions. Normoxia promoted higher expression of the markers typical of the chondrocyte phenotype (*ACAN*, *SOX-9*, *COL2A1*, *COL2A*, and *COL2B*), but also enhanced also the hypertrophic chondrocyte markers. Indeed, chondrogenic differentiation of hUCB-MSCs was associated with an up-regulation of type X collagen and *MMP-13*, mainly in conditions including TGF-β1. Our results are consistent with other studies that have found that in the presence of TGF-βs, cells increase the expression of genes characteristic of chondrocyte hypertrophy, including type X collagen and *MMP-13*, and the activity of alkaline phosphatase [8,24,47]. These gene expression patterns suggest that during chondrogenic differentiation MSCs may progress to the hypertrophic stage, observed during endochondral ossification and skeletal development or under pathological conditions, such as osteoarthritis (OA).

In the presence of both BMP-2 and TGF-β1 there was a stronger type X collagen expression compared to BMP-2 or TGF-β1, regardless of hypoxia. Chondrogenesis involves several steps (condensation, differentiation and maturation into chondrocytes) which require the action of specific growth factors in a spatio-temporal manner [48]. In this study, the addition of both growth factors allows them to be continuously available during the different required stages of differentiation. Determining the concentration and the exposure time of growth factors could be a way to prevent the induction of the hypertrophic chondrocytes phenotype during differentiation.

Moreover, one study demonstrated that in vivo ectopic implantation of human BM-MSC cartilage constructs obtained after seven weeks of chondrogenic differentiation in vitro in the presence of TGF-β3, leads to ECM mineralization, whereas implantation of differentiated articular chondrocytes maintains their phenotype [24]. To avoid mineralization in vivo in *nude* mice, Liu et al. suggested that the failure to maintain the phenotype of induced human BM-MSCs in subcutaneous location is due to insufficient chondrogenic differentiation in vitro and that completely differentiated MSCs can retain their chondrocyte phenotype to form stable ectopic cartilage [49]. Chondrogenic differentiation in vitro for 12 weeks stabilizes the differentiation into chondrocytes and prevents the calcification of the ECM after implantation in mice [49], suggesting that a fully differentiated chondrocyte phenotype must be acquired in vitro in hBM-MSCs in order to obtain a stable chondrogenesis in vivo. Surprisingly, we showed that, after two weeks of hUCB-MSCs chondrogenic differentiation in vitro in normoxia, cells maintained their phenotype and did not go into terminal differentiation and mineralize the ECM after ectopic implantation [6]. However, this neo-formed tissue may not be a perfect hyaline cartilage since some type I collagen staining, although weak, persists. An additional week of differentiation or a siRNA strategy may help stabilize the chondrocyte phenotype before implantation. For instance, our team has successfully used siRNAs to inhibit *COL1A1* in human articular chondrocytes [13].

In our experimental conditions, treatment of hUCB-MSCs with BMP-2 in hypoxia led to the lowest mRNA levels of hypertrophy markers. BMP-2 has been reported to induce hypertrophy of chondrocytes differentiated from hBM-MSCs and of MSCs of synovial origin [44,50]. However, the low BMP-2 concentration used in this study induced a lower expression of hypertrophy markers than other conditions with growth factors and counteracted the effect of TGF-β1 on *MMP-13* and HtrA1 expression when both growth factors are used. The expression of HtrA1 was induced at the protein level, under treatment with TGF-β1 and in the basal culture medium without growth factors. Up-regulation of HtrA1 expression in chondrocytes by TGF-β1 has been previously demonstrated [51,52]. For example, Xu et al. found that TGF-β1 induced the expression of HtrA1 in chondrocytes by phosphorylation of Smad 2/3 [52]. Our results suggest that the induction of HtrA1 expression in the absence of exogenous TGF-β1, may be due to the endogenous production of TGF-β1 by cells when they are cultured in a 3D environment, as found for the induction of typical pre-chondrocyte markers in 3D culture without addition of growth factors. These findings suggest a down-regulation of BMP-2 on the expression of HtrA1, which may be induced by the TGF-β1 treatment. Similarly, BMP-2 alone or in combination with TGF-β1, negatively regulated the expression of *MMP-13* messengers during chondrogenic differentiation between 7 and 21 days. MMP-13 is a matrix metalloproteinase that appears to play a key role in OA progression by promoting the degradation of the cartilage extracellular matrix. The expression level of *MMP-13* is significantly higher in chondrocytes of late-stage OA cartilage compared with early OA or normal knee cartilage [53]. TGF-β1 alone strongly induced the expression of *MMP-13*, which confirms the terminal maturation induced by this treatment. A degradative pathway underlying articular cartilage degeneration has been proposed with the presence and possible involvement of HtrA1, Ddr2, and Mmp-13 [52,54]. 

The down-regulation of *MMP-13* and HtrA1 protein expression by BMP-2 suggests that the two signaling pathways interact during chondrogenesis. BMP-2 inhibits the signaling of TGF-β1, whereas TGF-β1 reinforces that of BMP-2. An explanation of the negative feedback of BMP-2 here is that it may induce the inhibitory Smad7 protein. If so, great caution must be used when using TGF-β1 alone for cartilage repair, especially for UCB-MSCs.

We also assessed the expression of *MMP-1*, which is not regulated by the growth factors used. Culture conditions containing dexamethasone (10^−7^ M) negatively regulate the expression of MMP-1 [55]. In our model, cells in the presence or absence of growth factors are grown with ICM medium containing dexamethasone at the same concentration as the study of Jakobsen et al. This may explain the very low expression of *MMP-1* regardless of the culture conditions in our experimental model.

### 3.3. Effects of Oxygen Tension during hUCB-MSCs Differentiation

The natural microenvironment or niche of stem cells and chondrocytes is characterized by low oxygen tension [56,57]. The response of MSCs to oxygen tension with regard to subsequent chondrogenic differentiation is controversial. On the one hand, some studies report that low oxygen tension is beneficial for chondrogenic differentiation of MSCs [25,26,27]. On the other hand, a decrease in chondrogenic differentiation of MSCs and in self-renewal has been observed at low oxygen tension [28,29]. Reduced oxygen tension enhances chondrogenic differentiation in vitro for equine UCB-MSCs in a membrane culture model [17]. Here, we showed that normoxia promotes higher expression of markers specific to the chondrocyte phenotype than does hypoxia. These different findings may be attributed to the MSC species or culture conditions. We also observed that hUCB-MSC proliferation in monolayers was slower under low oxygen conditions compared with 21% O_2_ [33]. Our data therefore suggest that low physiological oxygen cultures may improve the maintenance of the undifferentiated state of hUCB-MSCs as shown for other stem cells [56,58,59]. In addition, in the presence of growth factors, hUCB-MSCs in hypoxia may resist commitment to chondrogenic differentiation. Furthermore, the earlier initiation of chondrogenic differentiation in normoxia in comparison with hypoxia in our study may be due to the stabilization of hypoxia-inducible factor-1α (HIF-1α) in normoxia. Surprisingly, hUCB-MSCs can display normoxic stabilization of HIF-1α that is usually completely degraded by HIF prolyl 4-hydroxylases in the presence of high oxygen tension [60]. It is well established that HIF-1α *trans*-activates SOX-9, a key factor necessary for the expression of many markers specific to chondrocytes [25,34]. Thus, stable expression of HIF-1α seems to promote chondrocyte differentiation of hUCB-MSCs and their responsiveness to growth factors in normoxia.

Several teams have used hypoxia and 3D cultures in order to redifferentiate chondrocytes or limit hypertrophy of MSCs during chondrogenesis [12,30,61]. Hypoxia inhibits *Col10a1* expression via down-regulation of Runx2 activity by suppressing Smad and activating histone deacetylase 4 [62]. In this study, we observed decreased expression of chondrocyte hypertrophy markers related to hypoxia culture. Based on these lines of evidence, we hypothesize that oxygen tension is an active regulator of hypertrophic differentiation. To benefit from both oxygen conditions, we initiated the chondrogenic differentiation of UCB-MSCs in normoxia and then continued culture in hypoxia. This strategy led to higher expression of type II collagen compared with the hypoxia condition, along with a considerable decrease in type X collagen and *MMP-13* expression. However, to the best of our knowledge, no study has reported the successive application of normoxia and hypoxia during chondrogenic differentiation and this innovative approach has not been tested by other teams. Given the environment of the chondrocytes in vivo, incubation in hypoxia seems clearly essential before transplantation. In addition, in vivo chondrogenesis undergoes different oxygen tensions. For example, in rabbits, the oxygen tension of the growth plate varies between 2.7% and 7.6% [63], suggesting that oxygen gradients can participate in the physiological control mechanism that regulates the growth of the growth plate. In our study, we demonstrated that the chondrocyte differentiation in vitro can be controlled by manipulating the oxygen tension. Thus, during in vitro culture, it is necessary to place the cells in hypoxia to hinder the terminal differentiation of the cells and therefore reduce chondrocyte hypertrophy.

## 4. Materials and Methods

### 4.1. Isolation and Culture of hUCB-MSCs

hUCB-MSC samples were collected at the Obstetrics and Gynecology Unit (Femme-Enfant-Hématologie Department at the Centre Hospitalier Universitaire in Caen, France) from normal delivery cases with written informed consent from the mothers following the hospital’s human ethics committee guidelines. All experimental protocols were approved by the local ethics committee on research with human samples (Comité de Protection des Personnes Nord Ouest III, Caen, France; January 2009).

**Figure 7:**
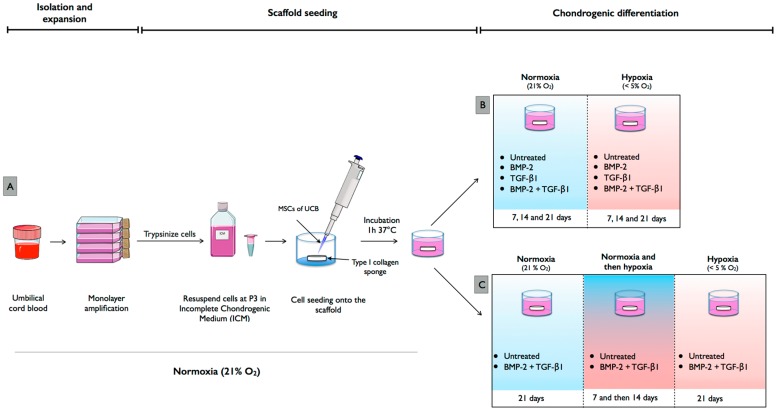
Isolation, amplification and chondrogenic differentiation of mesenchymal stem cells derived from umbilical cord blood. (**A**) Human umbilical cord blood (UCB) was collected after full-term normal-delivery births. Human mesenchymal stem cells (MSCs) derived from UCB samples were isolated by plastic adherence and expanded in tissue culture flasks until passage 3 in defined expansion medium containing fetal bovine serum under normoxia (21% O_2_). Thereafter, hUCB-MSCs were seeded at 5000 cells per collagen sponge. (**B**) hUCB-MSC-scaffold constructs were subsequently cultured under either normoxia or hypoxia (<5% O_2_) for 7, 14 and 21 days in serum-free chondrogenic medium containing bone morphogenetic protein-2 (BMP-2) or/and transforming growth factor-β1 (TGF-β1). (**C**) We also induced chondrogenic differentiation under two oxygen tensions. Differentiation was induced for seven days in normoxia, followed by 14 days in hypoxia. This figure was made with images available at Servier Medical Art (www.servier.fr).

hUCB units were collected in sterile flasks containing citrate phosphate dextrose anti-coagulant (Figure 7A). The blood was diluted in PBS in a 1:1 ratio and mononuclear cells were isolated by density gradient centrifugation at 400× *g* for 30 min in Ficoll-Paque^TM^ PREMIUM (at a density of 1.077 g/mL; GE Healthcare Bio-Sciences, Issaquah, WA, USA). Then, the collected mononuclear cells were washed once with PBS and plated in 25 cm^2^ tissue culture flasks in low glucose-Dulbecco’s modified Eagle medium (LG-DMEM; Invitrogen Life Technologies, Waltham, MA, USA) supplemented with 20% fetal calf serum (Invitrogen Life Technologies), 10^−7^ M dexamethasone (Sigma-Aldrich, St. Louis, MO, USA) and incubated at 37 °C in a humidified 5% CO_2_ atmosphere. After 48 h of incubation, non-adherent cells were removed and medium was replenished twice a week. About two to four weeks after plating, fibroblast-like adherent cells were observed and amplification was performed with the same media in the absence of dexamethasone. After the appearance of several colonies, cells were trypsinized (0.25% trypsin, Invitrogen Life Technologies), washed and, seeded at 5000 cells/cm^2^ (passage one, P1). Culture medium was replaced twice a week and at 80% confluency, cells were amplified up to P3. A cocktail of antibiotics and antifungals (penicillin: 100 IU/mL (Pan Pharma, Luitré, France), erythromycin: 100 μg/mL (Amdipharm, London, UK), fungizone: 0.25 mg/mL (Bristol-Myers Squibb, Rueil-Malmaison, France)) was included in the cell culture media used.

### 4.2. Human Articular Chondrocytes

Articular cartilage was harvested from macroscopically healthy zones of femoral heads of patients with osteoarthritis during total joint replacement surgery. Ethical approval for this study was obtained from the Ethics Committee of Caen (Comité de Protection des Personnes Nord Ouest III, Caen, France; January 2009). All patients signed an informed consent agreement for all samples used in this study. Articular cartilage was minced and chondrocytes were isolated by sequential enzymatic digestion as already described [12,13]. Briefly, small slices were treated with 2 mg/mL of type XIV protease (Sigma-Aldrich) for 45 min and then digested overnight with 1 mg/mL of type I collagenase (from *Clostridium histolyticum*; Invitrogen Life Technologies) at 37 °C. Cell suspension was filtered through a 70 μm mesh nylon membrane, centrifuged and rinsed at 200× *g* for 10 min to remove collagenase. Pelleted cells were re-suspended in TRIzol^®^ reagent (Invitrogen Life Technologies) and RNA extraction was carried out according to the manufacturer’s protocol. For Western blotting experiments, small slices of cartilage were ground in liquid nitrogen and protein extraction was performed with RIPA lysis buffer (150 mM NaCl, 50 mM Tris-HCl pH 7.4, 1% NP40, 0.25% DOC, 1 mM EDTA) containing protease inhibitors (1 mM Na_3_VO_4_, 5 mM NaF, 1 mM PMSF, 2 μg/mL aprotinin and 1 μg/mL leupeptin).

### 4.3. Chondrogenic Differentiation of hUCB-MSCs in Normoxia and Hypoxia

Chondrogenic differentiation was induced in 3D scaffold cultures (type I/III collagen sponges (2 mm thickness, 5 mm diameter), Symatèse Biomatériaux, Chaponost, France) in serum-free chondrogenic medium, which consists of incomplete chondrogenic medium (ICM) supplemented with 50 ng/mL BMP-2 (inductOs, Wyeth Europa Limited, Maidenhead, UK) and/or 10 ng/mL TGF-β1 (Miltenyi Biotec, Bergisch Gladbach, Germany) according to the following protocol. Briefly, hUCB-MSCs were trypsinized at P3 and 500,000 cells were seeded onto scaffolds in 96-well culture plates and incubated at 37 °C and 5% CO_2_ for 1 h. hUCB-MSC-scaffold constructs were subsequently transferred to 24-well plates in ICM with or without BMP-2 and/or TGF-β1. ICM consists of high glucose-DMEM (4.5 g/L) supplemented with 50 μg/mL ascorbic acid-2-phosphate (Sigma-Aldrich), 100 μg/mL sodium pyruvate (Invitrogen Life Technologies), 40 μg/mL proline (Fluka, Buchs, Switzerland), 1:100 dilution of Insulin-Transferrin-Selenium (Invitrogen) and 100 nM dexamethasone (Sigma). hUCB-MSCs seeded in collagen scaffolds in the presence of ICM without growth factors were considered as untreated controls in 3D culture. Cultures were maintained in normoxic or hypoxic conditions for 7, 14 or 21 days and medium was changed every 3 or 4 days for the duration of the experiment (Figure 7B). hUCB-MSCs expanded in culture flasks at P3 in LG-DMEM medium containing 20% fetal bovine serum in normoxia were used as undifferentiated hUCB-MSC controls.

### 4.4. Chondrogenic Differentiation with Modification of Oxygen Tension

Chondrogenic differentiation was induced in 3D scaffold cultures with or without BMP-2 (50 ng/mL), and TGF-β1 (10 ng/mL). We modified the oxygen conditions during chondrogenic differentiation (Figure 7C), by first inducing differentiation for seven days in normoxia followed by 14 days in hypoxia. hUCB-MSCs were induced to differentiate into chondrocytes for 21 days in either normoxia or hypoxia alone to serve as oxic controls.

### 4.5. RNA Extraction and RT-PCR 

Total RNAs were extracted after hUCB-MSCs chondrogenic differentiation in scaffolds or from monolayers of undifferentiated cells using TRIzol^®^ reagent according to the manufacturer’s instructions. One μg of total RNAs was used for reverse transcription with Moloney murine leukemia virus (MMLV, Invitrogen). qPCRs were performed on an Applied Biosystems 7700 Real-Time system using the TaqMan^®^ PCR Master Mix (Applied Biosystems, Foster City, CA, USA) for type IIB collagen cDNA and using Power SYBR Green PCR (Applied Biosystems) for the other cDNAs (*SOX-9*, *COL2A1*, *COL2A*, *ACAN*, *COL1A1*, *COL10A1*, *MMP-13*, *MMP-1*, *RUNX-2* and *OSTEOCALCIN*) as already published [12]. Gene expressions were normalized to an endogenous reference gene, *RPL13*, and compared with undifferentiated hUCB-MSCs cultured in monolayers. Relative gene expressions were calculated using the 2^−Δ*C*t^ method and expressed as the mean of triplicate samples.

### 4.6. Western Blotting

Scaffolds with differentiated cells or undifferentiated monolayer cells were washed with cold PBS, ground and total proteins were extracted by RIPA lysis buffer containing protease inhibitors. Protein concentration was evaluated by the Bradford procedure (Bio-Rad SA, Rosedale, New Zealand). Proteins lysates (20 μg) were separated on 10% SDS-PAGE gels and then transferred onto PVDF membranes (Millipore, Billerica, MA, USA). Non-specific binding sites of membranes were blocked with 10% non-fat milk powder in Tris-buffered saline containing 0.1% Tween (TBST, Sigma-Aldrich) for 1 h. Then, the blots were incubated overnight at 4 °C with anti-type I collagen (Novotec, 1:3000, Lyon, France), or anti-type II collagen (Novotec, 1:1500), or anti-type X collagen (Sigma, 1:5000), or rabbit anti-HtrA1 (Millipore, 1:2500). The membranes were also reacted with rabbit anti-GAPDH (Santa Cruz Biotechnology, Inc., 1:5000, Dallas, TX, USA) to verify equal loading. The next day, membranes were washed with TBST and, then incubated with HRP-conjugated goat anti-rabbit or anti-mouse IgG (Jackson ImmunoResearch, 1:5000, West Grove, PA, USA) for 1 h at room temperature with 3% milk in TBST. Bound antibodies were detected using a chemiluminescence assay (Western Lightning^®^ Plus-ECL, PerkinElmer, Inc., Waltham, MA, USA) and exposed to X-ray film for visualization.

### 4.7. Statistical Analysis

All experiments were repeated at least four times with different cell donors. Values were reported in box-plot graphs. All statistical analyses were done using Prism v5.0a (Graphpad, San Diego, CA, USA), and statistical analysis is detailed in the legend of each figure. *p*-values ≤ 0.05 were considered significant.

## 5. Conclusions

Our culture protocol promotes efficient chondrogenic differentiation, suggesting that UCB-MSCs can be a reliable source for cartilage tissue engineering. The addition of BMP-2 and TGF-β1 is the most efficient condition for inducing the differentiation of MSCs into chondrocytes. Finally, oxygen tension is a key factor in chondrogenic differentiation. Our data specifically suggest, for enhanced chondrocyte differentiation, that UBC-MSCs in in vitro 3D-culture should begin in normoxia to obtain efficient and quantitative chondrogenic differentiation before placing them in hypoxia to stabilize the chondrocyte phenotype before implantation. However, a better knowledge of the molecular mechanisms involved in the response to chondrogenic factors is a prerequisite for the use of MSCs in cartilage repair in the future. Improving the experimental protocols and attaining tissue repair requires full comprehension of the pathways involved in the chondrogenic differentiation of MSCs.
